# The Three-Dimensional Culture System with Matrigel and Neurotrophic Factors Preserves the Structure and Function of Spiral Ganglion Neuron* In Vitro*

**DOI:** 10.1155/2016/4280407

**Published:** 2016-01-06

**Authors:** Gaoying Sun, Wenwen Liu, Zhaomin Fan, Daogong Zhang, Yuechen Han, Lei Xu, Jieyu Qi, Shasha Zhang, Bradley T. Gao, Xiaohui Bai, Jianfeng Li, Renjie Chai, Haibo Wang

**Affiliations:** ^1^Otolaryngology-Head and Neck Surgery, Provincial Hospital Affiliated to Shandong University, Jinan 250022, China; ^2^Shandong Provincial Key Laboratory of Otology, Jinan 250022, China; ^3^Key Laboratory for Developmental Genes and Human Disease, Ministry of Education, Institute of Life Sciences, Southeast University, Nanjing 210096, China; ^4^Co-Innovation Center of Neuroregeneration, Nantong University, Nantong 226001, China; ^5^College of Medicine, The University of Tennessee Health Science Center, Memphis, TN 38163, USA

## Abstract

Whole organ culture of the spiral ganglion region is a resourceful model system facilitating manipulation and analysis of live sprial ganglion neurons (SGNs). Three-dimensional (3D) cultures have been demonstrated to have many biomedical applications, but the effect of 3D culture in maintaining the SGNs structure and function in explant culture remains uninvestigated. In this study, we used the matrigel to encapsulate the spiral ganglion region isolated from neonatal mice. First, we optimized the matrigel concentration for the 3D culture system and found the 3D culture system protected the SGNs against apoptosis, preserved the structure of spiral ganglion region, and promoted the sprouting and outgrowth of SGNs neurites. Next, we found the 3D culture system promoted growth cone growth as evidenced by a higher average number and a longer average length of filopodia and a larger growth cone area. 3D culture system also significantly elevated the synapse density of SGNs. Last, we found that the 3D culture system combined with neurotrophic factors had accumulated effects in promoting the neurites outgrowth compared with 3D culture or NFs treatment only groups. Together, we conclude that the 3D culture system preserves the structure and function of SGN in explant culture.

## 1. Introduction

In mammalian cochleae, spiral ganglion neurons (SGNs) are specialized bipolar neurons that transmit auditory information from ear to brain. SGNs originate from the cochleovestibular ganglion [1, 2] and then mature along with the specialization of hair cells (HCs) within the prosensory domain of the cochlea. Finally, the SGNs extend peripheral axons that couple to HCs at the ribbon synapse [3], through which the SGNs transmit auditory information from HCs to the central nervous system. Therefore, the survival of SGNs is indispensable for the preservation of hearing, and damage to SGNs exacerbates hearing loss [4]. However, the mechanisms underlying the stimulation and guidance of neurite outgrowth from SGNs to their targets are still unclear and are in need of further exploration.

The two traditional approaches of dissociated cell culture and conventional organotypic culture have been commonly used for culturing SGNs in previous research. Dissociated cell culture has been widely used to study the survival [5, 6] and neurite regeneration [7] of SGNs. Although the dissociated spiral ganglion cell culture contains all of the cell types in the spiral ganglion region and might also contain the soluble factors that are provided by glia cells (GCs) for the support of the SGNs, the cell-cell adhesions are disrupted and the physiological cell-cell interaction between SGNs and GCs is destroyed. SGNs are arranged in a three-dimensional (3D) structure in the cochlea* in vivo*; thus, it is necessary to maintain this environment if one wants to observe how SGNs act normally in biological processes and how they react to stress [3]. The conventional organotypic culture involves adhering the freshly dissected spiral ganglion organ onto the dish surface, and this type of culture is also called two-dimensional (2D) culture [8, 9]. Conventional 2D organotypic culture has been used to explore the effects of small molecules on the development of SGN neurites [9] and the changes of gene expression in response to injury and hypoxia [10] and so on. However, because it is unable to provide the physical scaffold needed for the outgrowth of SGNs, neurites extending from spiral ganglions under 2D conditions are very few and very short [11, 12], and this makes the 2D culture a less than ideal culture system to study the structure and function of SGNs. Therefore, the 3D culture system, which can encapsulate SGNs to mimic the normal 3D environment* in vivo*, is regarded as the ideal model to preserve the delicate structure and function of SGN explants in culture.

Among all kinds of 3D culture systems, matrigel, which is a reconstituted basement-membrane-like matrix (BM), has been successfully used as a 3D culture model in inner ear research. For instance, Edin et al. cultured human superior vestibular ganglions in 3D-matrigel* in vitro* to investigate inner ear neuron regeneration [13], and Spencer et al. have used matrigel as a scaffold for chicken cochlear cultures to develop an HC regeneration model [14]. These studies have demonstrated the bioactivity and tissue compatibility of matrigel in inner ear tissues. However, the effect of 3D-matrigel culture in maintaining the structure and function of SGNs in explant culture remains uninvestigated.

In this study, we succeeded in establishing the 3D culture system with matrigel to encapsulate the spiral ganglion region isolated from neonatal mice. This system was able to protect the SGNs against apoptosis, promote the growth of growth cone, boost the neurite sprouting and outgrowth, and elevate the synapse density of SGNs. When combined with neurotrophic factors, 3D system also has accumulated effect to significantly promote the neurites outgrowth. In conclusion, the 3D-matrigel culture system preserved the structure and function of SGN in short-term culture, which may be applicable to the* in vitro* study of the physiology and pathophysiology of the inner ear.

## 2. Materials and Methods

### 2.1. Animals and SGN Dissection

All experiments were approved by the Animal Care Committee of Shandong University, China, on the care and use of Laboratory Animal for Research Purposes. Postnatal 3-day-old (P3) C57BL/6 mice were purchased from the Animal Center of Shandong University (Jinan, China).

The dissection procedures were performed as described in previous studies with slight modifications [9, 15]. P3 C57BL/6 mice were decapitated and skulls were opened midsagittally with a pair of small surgical scissors. Immediately, temporal bones of two sides were cut off and placed into sterile Hank's Balanced Salt Solution (Hyclone, USA) on a flat ice pack. The cochlear capsule was removed by fine forceps to expose the membranous labyrinth under a dissecting microscope. The stria vacularis and the organ of Corti were removed and the middle turn was chosen to keep sampling consistence between groups. After discard of the modiolus, the remnant SGN bulk was cut into 4 equal portions about 200 *μ*m in diameter each.

### 2.2. Organotypic Cell Culture

There were two different media used in the study; the primary growth medium (PGM) consists of 10% fetal bovine serum (Gibco, USA) and 50 *μ*g/mL ampicillin in DMEM/F12. The full medium (FM) was supplemented with N2 (1 : 100, Invitrogen, USA), B27 (1 : 50, Invitrogen), epidermal growth factor (EGF, 20 ng/mL, Sigma, USA), basic fibroblast growth factor (bFGF, 10 ng/mL, Sigma), insulin-like growth factor-1 (IGF-1, 50 ng/mL, Sigma), heparan sulfate (50 ng/mL, Sigma), and ampicillin (50 *μ*g/mL, Sigma) in DMEM/F12 (Gibco) and should be used within two weeks.

Isolated SGN explants were adhered onto a 10 mm glass coverslip precoated with CellTak (BD Biosciences, USA) and cultured in PGM in 4-well dish (Greiner Bio-One, Germany) overnight without any treatments. Then, the next day, the media were changed into FM combined with different treatments as follows.

For 2D culture, explants were kept in FM without any additives, as the uniform control of the other treated groups.

For 3D culture, the concentrations of matrigel added in FM include 2%, 10%, 20%, and 50%. Correspondingly, 2 *μ*L, 10 *μ*L, 20 *μ*L, or 50 *μ*L ice-cold matrigel was mixed together with 98 *μ*L, 90 *μ*L, 80 *μ*L, or 50 *μ*L FM and dropped on to the tissue explants directly. The gelation was initiated to solidify immediately at room temperature and was completed 10 min later. Then, the culture dish was transferred to the incubator.

For neurotrophic factor- (NF-) treated groups, 10 ng/mL BDNF (R&D Systems, USA) and 10 ng/mL NT3 (R&D Systems, USA) were added in the 2D culture or 20% matrigel 3D culture system, which were regarded as the 2D-NF or 3D-NF group, respectively.

All treated explants were then cultured for 48 h or 7 days at 37°C, 5% CO_2_, and 95% humidity.

### 2.3. Immunostaining

After organotypic culture, SGN explants were incubated in ice-cold PBS at 4°C for 30 min and washed twice with PBS. In order to keep the architecture structure of SGNs, the coated matrigel was carefully removed except that the surrounded cultured tissue, followed by the fixation with 4% paraformaldehyde and permeabilization with 1% TritonX-100 in PBS (Sigma), samples were immersed in blocking solution (0.1% TritonX-100, 8% donkey serum, 1% bovine serum albumin, and 0.02% sodium azide in PBS) at room temperature for 1 h. Then, samples were incubated with different primary antibodies: *β*-tubulin (1 : 1000, Neuromics, USA), neurofilament (1 : 1000, Millipore, USA), and synaptophysin (1 : 1000, Millipore), diluted in blocking solution, respectively, at 4°C overnight. The next day, tissues were incubated with FITC-conjugated or TRITC-conjugated (1 : 1000, Invitrogen) secondary antibody along with DAPI (1 : 800, Sigma-Aldrich) or phalloidin (1 : 1000, Sigma-Aldrich) in 0.1% TritonX-100 and 1% BSA in PBS at room temperature for 1 h. Then, the coverslips were mounted and observed under a laser scanning confocal microscope (Leica, Germany).

### 2.4. Terminal Deoxynucleotidyl Transferase dUNT Nick End Labeling (TUNEL) Detection

Cell apoptosis was studied by DNA fragmentation with a TUNEL staining kit (Click-iT Plus TUNEL Assay for* In Situ* Apoptosis Detection, Invitrogen) according to the manufacturer's instruction. The nucleus was stained with DAPI, and the explants were evaluated using the confocal microscopy (Leica, Germany).

### 2.5. RNA Extraction and Real-Time Polymerase Chain Reaction (RT-PCR)

After being cultured in the 3D-matrigel for 48 h, SGN explants were firstly incubated in ice-cold PBS at 4°C for 30 min and the coated matrigel turned into liquid form; then, the explant was washed three times with ice-cold PBS to remove the matrigel clearly. Then, 20 explants were collected into TRIzol (Life Technologies, USA) to obtain the total RNA following the manufacturer's instructions. The RNA concentration was measured with a Bio-Rad spectrophotometer. cDNA was synthesized from 1 *μ*g total RNA by reverse transcription using the Revert Aid First Strand cDNA Synthesis Kit (Thermo Scientific, USA) following the manufacturer's protocols. Quantitative real-time PCR (RT-PCR) was performed using SYBR Premix Ex Taq (TaKaRa, Japan). GAPDH was used as housekeeping gene. Each 25 *μ*L PCR reaction mixture contained 12.5 *μ*L 2X SYBR Green PCR Master Mix, 0.5 *μ*L forward primer (10 *μ*M), 0.5 *μ*L reverse primer (10 *μ*M), 2 *μ*L template, and 9.5 *μ*L sterilized distilled water. Each group contained three samples and each PCR was carried out in triplicate. The conditions of PCR were as follows: 95°C for 10 min followed by 40 cycles of 95°C for 15 s, 60°C for 15 s and 72°C for 30 s followed by dissociation at 95°C for 15 s, 60°C for 30 s, and 95°C for 15 s. All data were analyzed using the Eppendorf Realplex 2. PCR primers for the genes were listed in Table 1.

### 2.6. Image Analysis

Confocal images of SGNs were taken using a Leica SPE confocal fluorescence microscope (Leica). Z-stack images were taken at 0.2 *μ*m intervals to span the samples. The number, length, height, and area of the neurite outgrowth were evaluated separately. As shown in the schema diagram of Figures 2(a)–2(d), the number of SGN neurites directly extended from each explant was counted, the length of neurite was measured from the base of the neurite at the explant edge to its farthest end, and the distance between the upper and the lower planes of stained neurites was regarded as the average height of neurites in SGN explant. As for the area measurement, all the far ends of neurites of each explant were connected to form an irregular closed shape, whose area was calculated by Image J software.

### 2.7. Statistical Analysis

For each processing condition, at least three individual experiments were conducted. Data were presented as mean ± SEM (standard error of the mean), and comparisons between groups were tested by Student's *t*-test. *p* < 0.05 was considered statistically significant.

## 3. Results

### 3.1. Establish the 3D Culture System and Optimize the Concentration of Matrigel

**Figure 1:**
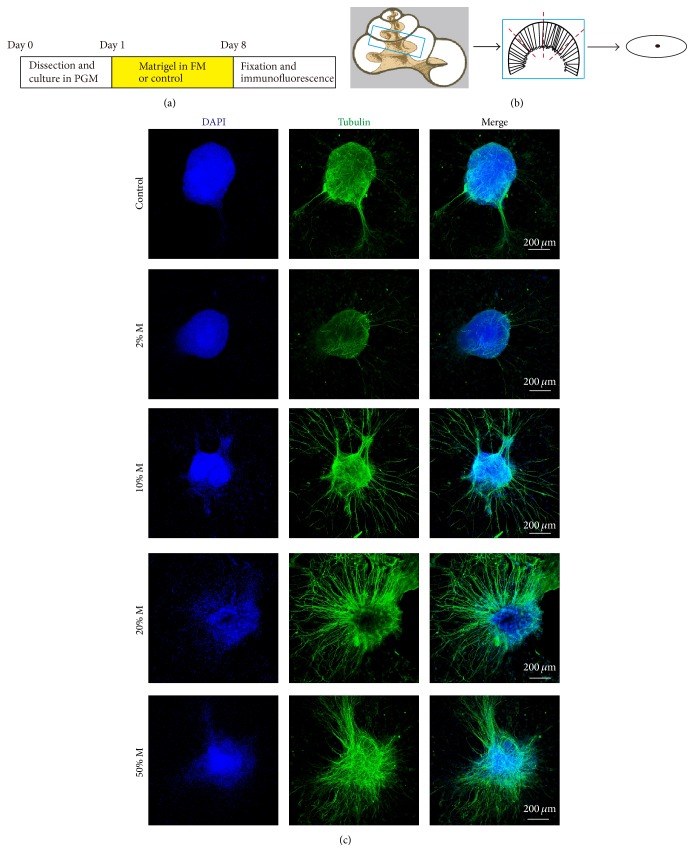
Immunofluorescence imaging of SGN explants cultured with different concentrations of matrigel. (a) The diagram of the assay. (b) The schematic of dissection and culture of SGN explants from neonatal mice cochleae. The middle turn of P3 cochlea was dissected out, the stria vacularis, the organ of Corti, and modiolus were discarded, and remnant SGN bulk was cut into four equal pieces, attached to coverslips, and cultured. (c) Representative SGN explants stained with DAPI (blue) and anti-*β*-tubulin (green) antibody for each experimental condition.

**Figure 2:**
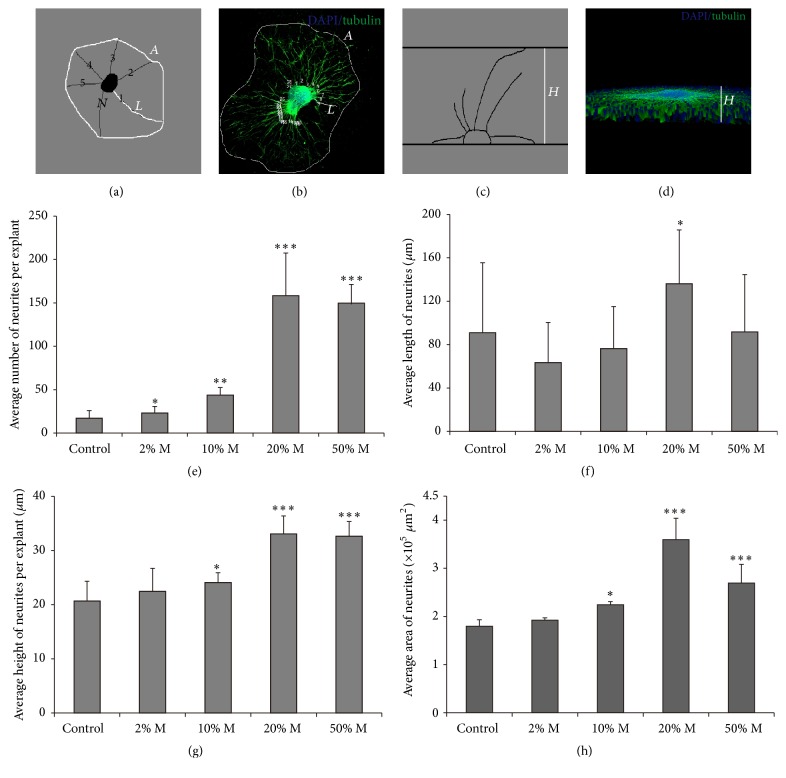
3D-matrigel culture promoted neurite outgrowth of SGN explants. (a) The planar characteristics of SGN explants, the number (*N*), the length (*L*), and the area (*A*) of neurites, were measured. (b) SGN explant as the illustration for number, length, and area of neurites. (c) The schematic of the height (*H*) of neurites in SGN explant. (d) SGN explant as the illustration for the height of neurites. (e–h) Quantifications of the average number, length, height, and area of neurites. ^*∗*^ *p* < 0.05, ^*∗∗*^ *p* < 0.01, and ^*∗∗∗*^ *p* < 0.001.

After being cultured* in vitro* for 7 days, explants were immunostained with the SGNs marker *β*-tubulin and observed under the confocal microscope. As illustrated in Figure 1(c), in the 2D cultured group, only several neurite outgrowths were extended from the explant, and with limited length. The 3D culture with matrigel promoted the growth of SGN neurites remarkably (Figure 1(c)). We compared four parameters of the neurite outgrowth (the number, length, height, and area) to analyze the effect of 3D culture statistically, and as shown in Figures 2(e)–2(h), 2% matrigel did not increase the growth of neurite compared with the 2D culture control group, except for slight increase in the number of neurites (data not shown here), while 10% matrigel culture significantly promoted the number, height, and area of the neurites to 2.57-fold, 1.17-fold, and 1.25-fold (*p* < 0.01, *p* < 0.05, and *p* < 0.05) that in control group. Remarkably, 20% matrigel dramatically increased all four parameters of SGN neurite outgrowth, as the average number, length, height, and area of neurite have been raised to 9.31-fold, 1.50-fold, 1.60-fold, and 2.00-fold of control (*p* < 0.001, *p* < 0.05, *p* < 0.001, and *p* < 0.001, resp.), while 50% matrigel increased the number, height, and area to 8.87-fold, 1.58-fold, and 1.50-fold (*p* < 0.001, *p* < 0.001, and *p* < 0.001), but the length of neurites has no significant difference compared with the control group. Thus, these data suggested that 20% matrigel could benefit the cultured SGN explants the most as they grew much more and longer neurites, as well as extended larger height and area, and was chosen for the 3D culture in the following experiments.

### 3.2. 3D Culture Protects the SGN Explants against Apoptosis

**Figure 3:**
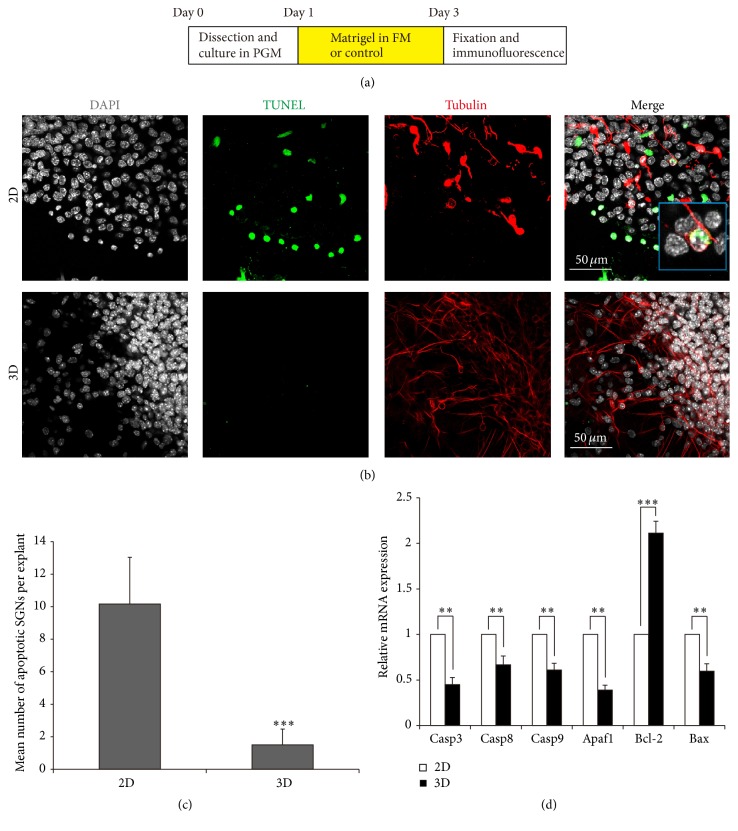
3D culture protected SGN explants from apoptosis. (a) The diagram of the assay. (b) Apoptotic SGNs (white arrow) were observed in 2D culture, characterized by DAPI (grey), TUNEL (green), and *β*-tubulin (red) costained. (c) Compared with 2D culture, 3D culture reduced the average number of apoptotic SGNs. ^*∗∗∗*^ *p* < 0.001. (d) 3D culture regulated the mRNA expressions of apoptotic genes. ^*∗∗*^ *p* < 0.01 and ^*∗∗∗*^ *p* < 0.001.

Taking advantage of TUNEL assay to detect the apoptosis, we observed significant amount of apoptotic SGNs in 2D group after being cultured for 48 h* in vitro* and found that the 3D culture system had significant protective effect against apoptosis on the SGN explants. As evidenced in Figure 3, apoptotic SGNs showed an abnormal or irregular morphology such as the breakage of nerve fibers, shrinkage of neurons, and nuclear condensation. The TUNEL assay confirmed that 2D culture caused apoptotic death in SGNs as there were 10.17 ± 2.87 TUNEL-*β*-tubulin double positive neurons out of one explant. However, very few TUNEL-*β*-tubulin double positive cells were found in 3D culture system with 20% matrigel (Figure 3(c)), suggesting that the 3D condition could be helpful in reducing apoptosis and surviving longer for the SGN explants.

Furthermore, RT-PCR data showed that SGN explants cultured in 3D-matrigel system had significantly lower expression of proapoptotic genes Casp8, Casp9, Casp3, Apaf1, and Bax compared to the 2D group (*p* < 0.01, Figure 3(d)), while they had significantly higher mRNA expression of antiapoptotic gene Bcl-2 in the 3D group (*p* < 0.001, Figure 3(d)). These data demonstrated that 3D culture with matrigel could regulate the expression of apoptosis related genes and protected the SGN explants from apoptosis.

### 3.3. 3D Culture Preserves the Delicate Structure of SGN Explants

**Figure 4:**
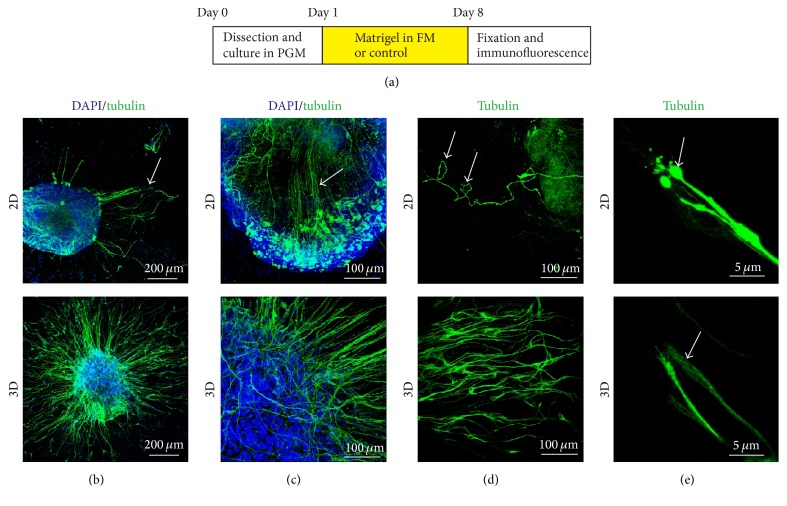
3D culture preserved the delicate structure of neurites in SGN explants. (a) The diagram of the assay. Fasciculation (b), reversal (c), curling (d), and swelling (e) observed on SGN explants in 2D group. DAPI, blue; *β*-tubulin, green.

We found some abnormal growth patterns of SGN neurites in the 2D cultured group after being cultured for 7 days* in vitro*, such as reversal, fasciculation, curling, and swelling, which were absent in the 3D cultured groups.  Figure 4(c) showed the reversal performance of the neurites, instead of extending out from the edge of explant and developing in a radial structure as in the 3D group, some neurites grew back to the original explant and in a disordered model. Sometimes, neurites changed their radial growth direction from the edge of the explant and clumped into fascicles (Figure 4(b)), some curved in the process (Figure 4(d)), which were regarded as fasciculation and curling, respectively. Moreover, a prominent swelling could be seen at the termination of some neurites in 2D culture group (Figure 4(e)). However, none of these patterns was found in the 3D cultured groups. These results indicated that 3D culture preserved the delicate structure in the extension of neurites of SGN explants.

### 3.4. 3D Culture Promotes the Growth of Growth Cones

**Figure 5:**
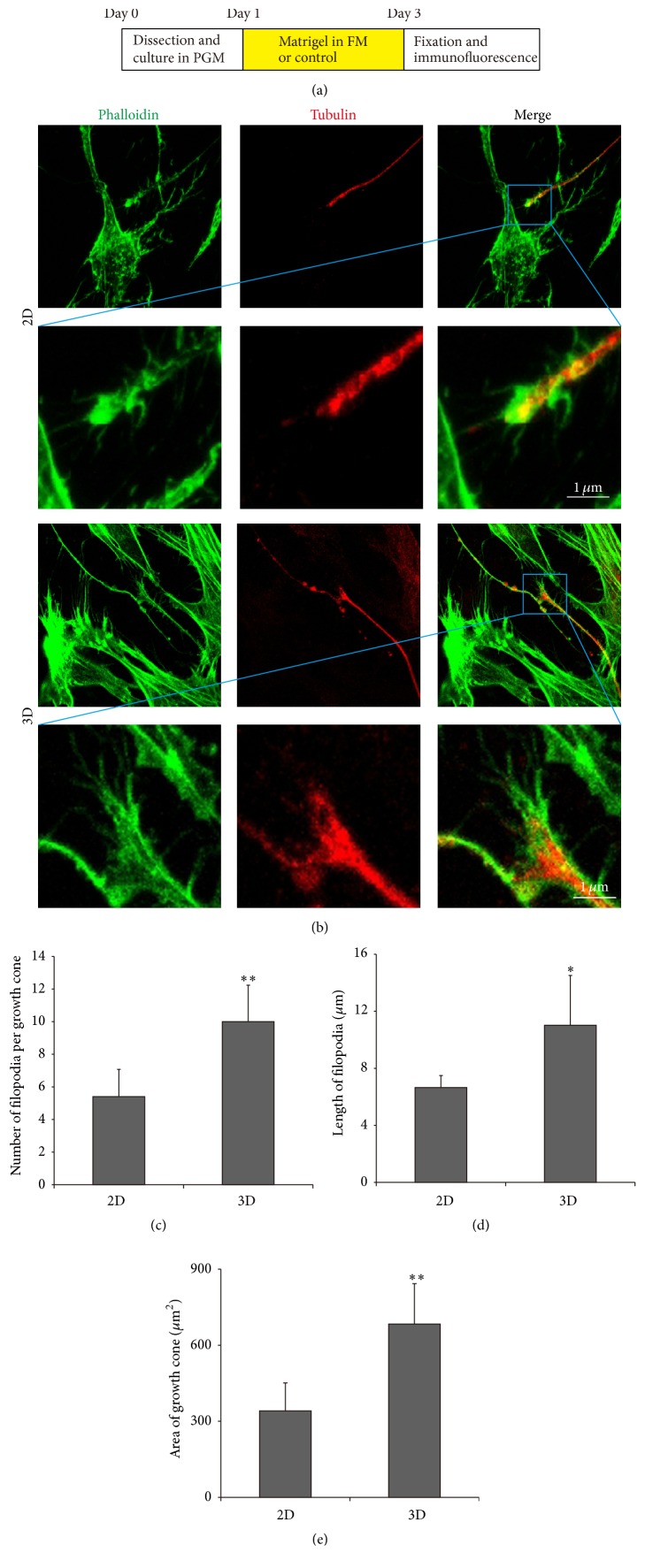
3D culture affected the growth of growth cones. (a) The diagram of the assay. (b) Morphology of the SGNs growth cone cultured in 2D and 3D systems. Phalloidin, green; *β*-tubulin, red. (c) Quantification of the average number of filopodia per growth cone. (d) Quantification of the average length of filopodia from the edge of the growth cone to tips of each filopodia. (e) Quantification of the average area per growth cone. ^*∗*^ *p* < 0.05 and ^*∗∗*^ *p* < 0.01.

Next we investigated the effect of 3D culture on the SGN growth cones by comparing the number, length of filopodia, and the area of growth cone after being cultured for 48 h* in vitro*. We stained the cultured SGN with phalloidin and *β*-tubulin and found the average number of filopodia emerging from the growth cones was greater when cultured in 3D culture system compared to the 2D group (*p* < 0.01, Figure 5(c)). Similarly, the average filopodia length from the tips of individual filopodia to the edge of the growth cone was longer (*p* < 0.05, Figure 5(d)), and the growth cone area of neurites was larger when cultured under 3D condition than those under 2D system (*p* < 0.01, Figure 5(e)). Altogether, the 3D culture condition facilitated the growth of SGN growth cones and the development of filopodia compared to the 2D group.

### 3.5. 3D Culture Enhances the Sprouting of Neurites in SGN Explants

**Figure 6:**
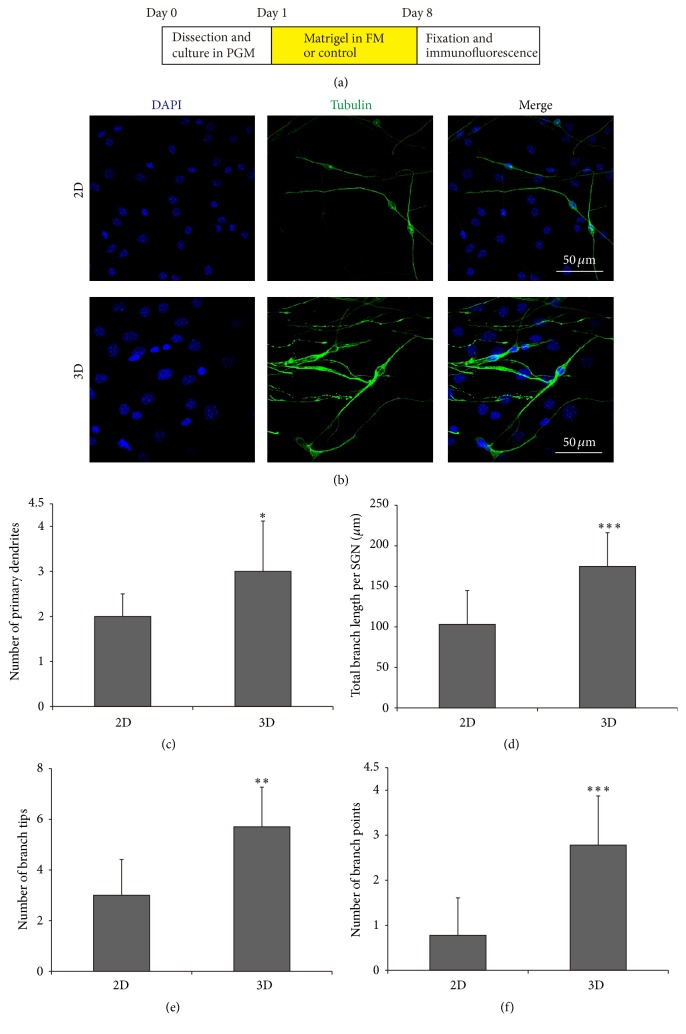
3D culture improved the sprouting of neurites in SGN explants. (a) The diagram of the assay. (b) Representative images of SGNs explants cultured in 2D and 3D systems. DAPI, blue; *β*-tubulin, green. (c–f) Quantifications of the average number of primary dendrites, total branch length, number of branch tips, and number of branch points in 2D and 3D systems. ^*∗*^ *p* < 0.05, ^*∗∗*^ *p* < 0.01, and ^*∗∗∗*^ *p* < 0.001.

The sprouting of neurites in SGN explants was measured after being cultured for 7 days* in vitro*, and Figure 6(b) illustrated that more dendrites were extended from the cell bodies of SGNs in explants in 3D culture systems than in the 2D group (Figure 6(b)). Statistical analysis showed that the number of primary dendrites, the total branch length, the number of branch tips, and the number of branch points were all elevated significantly in 3D group compared to the 2D culture group (Figures 6(c)–6(f), *p* < 0.05, *p* < 0.001, *p* < 0.01, and *p* < 0.001, resp.), suggesting that 3D culture promoted neurite sprouting and outgrowth of SGN explants.

### 3.6. 3D Culture Elevates Synapse Density in SGN Explants

**Figure 7:**
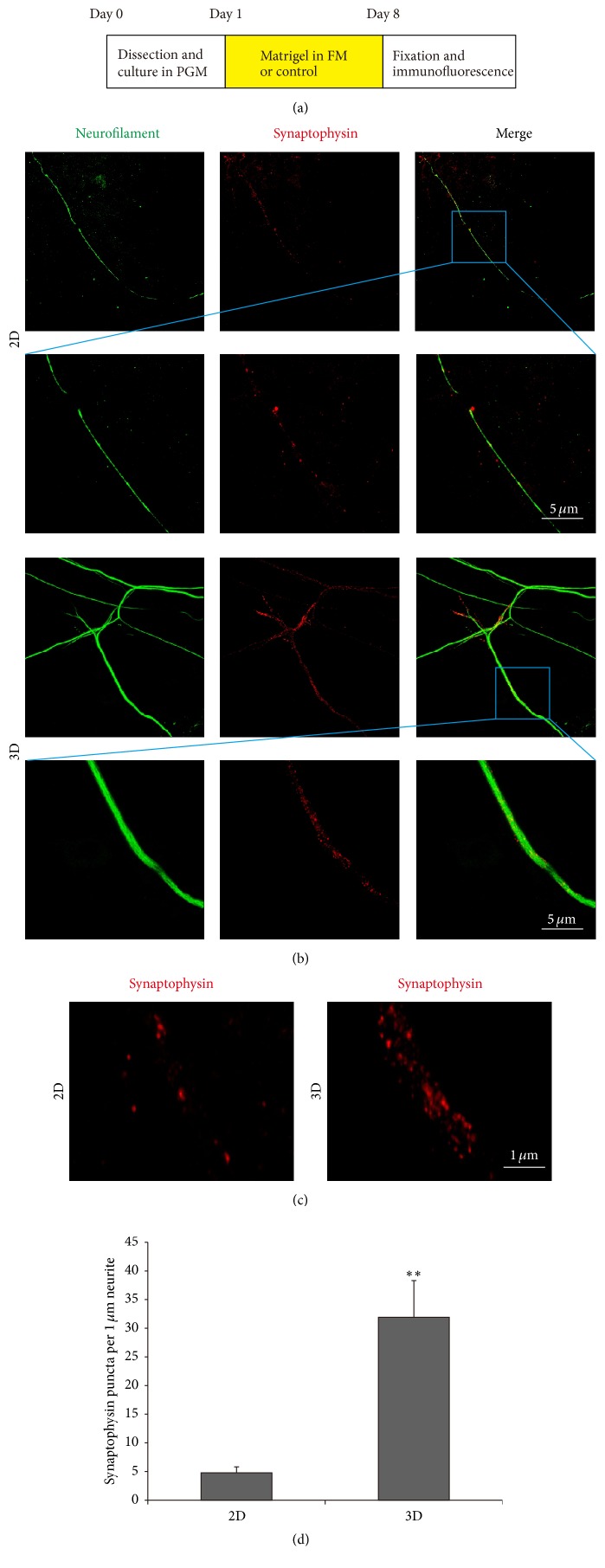
3D culture elevated synapse density in SGN explants. (a) The diagram of the assay. (b) Representative images of SGNs explants immunostained with neurofilament (green) and synaptophysin (red) cultured in 2D and 3D systems. (c) Representative images in high magnification of synaptophysin (red) immunostained SGN explants in 2D and 3D systems. (d) Quantification of the number of synapse puncta in 2D and 3D systems. ^*∗∗*^ *p* < 0.01.

To further investigate the effect of 3D culture on the function of organotypic SGNs, we also detected the synaptophysin expression in neurites to analyze the synapse density. Synaptophysin is a membrane protein specific to synaptic vesicles and correlates directly with the presence of neurotransmitter [16, 17]. Therefore, it also serves as a specific presynapse marker. Representative images after culture in 20% matrigel system for 7 days were shown in Figure 7. 3D system significantly elevated the synapse density during culture compared to 2D controls (Figures 7(b) and 7(c)). Statistical analysis proved that synaptophysin puncta density was significantly increased in 3D system compared to 2D group (*p* < 0.01, Figure 7(d)), indicating that 3D system promoted synapse maturation and synaptic plasticity in SGN explant culture.

### 3.7. Synergistic Influence of Matrigel 3D System with BDNF and NT3 on Neurite Outgrowth

**Figure 8:**
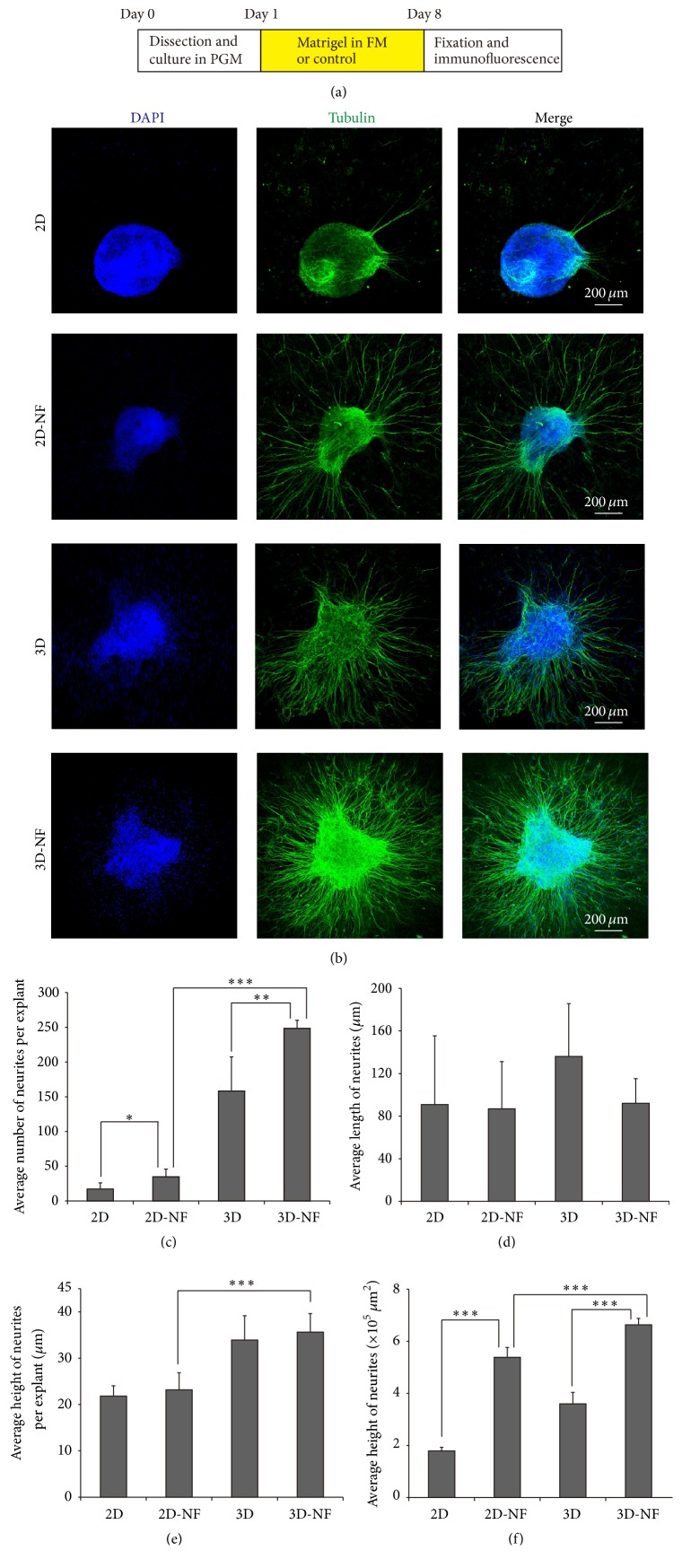
Synergistic influence of matrigel with BDNF and NT3 on neurite outgrowth. (a) The diagram of the assay. (b) Representative staining of neurites observed on SGN explants treated with 2D 2D-NF, 3D, and 3D-NF. DAPI (blue), *β*-tubulin (green). (c–f) Quantifications of the average number, length, height, and area of neurites in treated groups. ^*∗*^ *p* < 0.05, ^*∗∗*^ *p* < 0.01, and ^*∗∗∗*^ *p* < 0.001.

In the auditory system, diverse factors are reported to play important roles in the development and morphology of SGNs, including brain-derived neurotrophic factor (BDNF) and neurotrophin-3 (NT3). Based on the above findings that 3D-matrigel culture system could promote the outgrowth of SGNs, we further explored the combined effects of 3D culture system together with NFs on SGN explants after 7 days in culture. As shown in Figure 8, treatment with BDNF and NT3 in 3D system significantly increased the number and area of neurites compared to NFs-only and 3D-only groups (*p* < 0.01, *p* < 0.001, *p* < 0.001, and *p* < 0.001, resp.), but not the length of SGNs. Moreover, the number, height, and area of neurites were significantly enhanced in the 3D-NF group when compared with the 2D-NF group (*p* < 0.001, *p* < 0.001, and *p* < 0.001, resp.). Altogether, these results demonstrated that 3D culture system combined with NFs has accumulated effects in promoting the neurites outgrowth, indicating the BDNF and NT3 have synergistic influence on the neurite outgrowth of SGN explants, especially under the 3D-matrigel culture condition.

## 4. Discussion

Whole-organ culture of the organ of Corti has been used as the popular model to study both the biology and physiology of cochlear cell types and also the pathologic processes affecting them. Specifically, the 2D culture has been used to examine the effects of growth factors on SGNs [18–21]. Although this method is simple and provides a viable culture for SGN explant, it does not adequately preserve the natural complex 3D structure of the cochlea. In this study, we used the matrigel to construct a 3D culture system to wrap up the SGN tissue bulk* in vitro.* To our knowledge, this is the first report about the 3D culture system that supplied physical support for the murine SGN explant and maintained its physiological structure and function.

Matrigel is a commercially available analog of physiological BM, which has been used to make different kinds of 3D culture models [22–24]. In the present study, the 3D-matrigel culture system promoted the neurite outgrowth of SGN explant significantly compared with the 2D group; not only were the planar characteristics, including the length and number of SGNs neurites, increased, but also the spatial attributes, such as height and area of the neurite outgrowth, were enlarged remarkably, and the latter factors were thought to be more important for maintaining the space structure of spiral ganglion region. Meanwhile, the 3D culture preserved the delicate structure of SGN explants as no abnormal growth patterns of SGN neurites were observed in the 3D cultured group. Thus, these results suggested that the culture system built by matrigel could be used to mimic the 3D organization of SGNs under physiologic conditions. Furthermore, our results also revealed that the effect on neurite outgrowth of SGN explants was dependent on concentrations of matrigel, and the 20% matrigel was considered as the most effective concentration as it promoted all the four parameters tested in the experiment. One possible reason for this is that the physical property of matrigel is related to its concentration: in our study, 20% matrigel became jelly-like substance at 37°C, while 2% matrigel was still liquid and cannot provide a 3D condition, or 50% matrigel was too thick which might have limited the tension and spreading of SGN neurites.

Previous studies reported that SGNs in 2D cultured explants inevitably degenerate in a few days [25], and 3D system is thought to be the potential way to improve the culture condition and to preserve the structure and function of cultured explants. In our research, we also observed significant amount of apoptotic SGNs in the 2D culture group as evidenced by TUNEL assay and RT-PCR results (Figure 3), while in 3D culture system very few apoptotic SGNs were observed, significant lower expression of proapoptotic genes and higher expression of antiapoptotic gene were detected (Figure 3(c)), suggesting that the 3D condition significantly reduced the apoptosis and enhanced the survival of SGN explants. These findings therefore allow us to speculate that the 3D environment not only provided the scaffold to support the SGN explant but also conducted the protective action by building up a 3D structure to provide larger surface area and better nutrient transmission. For the matrigel 3D culture system, the major component of matrigel is laminin, but the culture media also contain EGF, IGF-1, bFGF, and other growth factors. Since the 3D structure system provided larger surface and better nutrient transmission, the growth factors in the culture media could be better taken inside into the cultured organs. It has been reported that the FGF participated in SGN development and support their postnatal survival following differentiation [26, 27]; the FGF/FGF receptor signaling affected the C-Jun-N-terminal kinase- or caspase-dependent pathways to alter apoptosis in cochlea [28]. EGF receptor has been found to be expressed in both the neonatal and adult mice spiral ganglion [29, 30], and IGF-1-deficient cochlear neurons showed increased caspase-3-mediated apoptosis [31]. Therefore, the better uptake of these growth factors in 3D-matrigel system might regulate the above signaling pathways in SGNs and might be responsible for the downregulation of proapoptotic factors and the increased survival ratio, but this still needs to be further explored in the future.

Growth cone is dynamic, actin-supported extension of a developing neurite seeking its synaptic target. Filopodia are the dominant structures in growth cones. In the present study, after being cultured in the 3D-matrigel culture system, the number, length, and area of filopodia in growth cones were enhanced significantly compared to the 2D culture group (Figure 5). In addition, the number of primary dendrites, the total branch length, the number of branch tips, and the number of branch points were also significantly elevated in 3D-matrigel culture system. Altogether, these results suggest that the 3D culture condition facilitated the growth of growth cones, the development of filopodia, and the neurite sprouting and outgrowth compared to the 2D system.

In mammals, sound information is detected by mechanosensitive HCs and transmitted to SGNs through ribbon synapses. By examining somatic presynaptic and postsynaptic protein content in SGNs new insights into the functional organization of the auditory primary afferents can be provided. In our research, synaptophysin was used as the presynapse marker, and the expression was enhanced in the 3D culture group compared to the 2D group, demonstrating that the synapse density was significantly improved under the 3D culture condition, which further suggests that the 3D-matrigel culture system has the potential to promote the synapse maturation and synaptic plasticity of SGNs.

In the auditory system, BDNF and NT3 are predominant NFs, which have distinct functions in the development and maintenance of SGNs in developing inner ear [32]. In our study, treatment with BDNF and NT3 to SGN explants increased the number and area of neurites both in 2D and 3D systems compared with those of the NF-only and 3D-only groups separately. More importantly, the number, height, and area of neurites were significantly enhanced in the 3D-NF group when compared with the 2D-NF group, indicating that 3D culture system has accumulated effects in promoting the neurites outgrowth when combined with NFs. However, the length of neurite was not affected by the administration of BDNF and NT3 in both 2D and 3D systems, which is consistent with the previous studies in 2D culture system [8].

Taken together, 3D culture system with matrigel exhibited excellent biocompatibility and promoted the survival, structure, outgrowth, and function of SGNs throughout the culture period compared to the 2D culture. The present study highlighted the potential of matrigel as a 3D culture system applied to the* in vitro* study of the physiology and pathophysiology of the inner ear.

## Figures and Tables

**Table 1:** PCR primer sequences used in the experiments.

Gene	Forward sequence	Reverse sequence
Casp8	GCTGTATCCTATCCCACG	TCATCAGGCACTCCTTT
Casp9	GGACCGTGACAAACTTGAGC	TCTCCATCAAAGCCGTGACC
Casp3	GGAGCAGCTTTGTGTGTGTG	CTTTCCAGTCAGACTCCGGC
Apaf1	TGTGTGAAGGTGGAGTCAAGG	CCTCTGGGGTTTCTGCTGAA
Bcl-2	TGACTTCTCTCGTCGCTACCG	GTGAAGGGCGTCAGGTGCAG
Bax	CGTGGTTGCCCTCTTCTACT	TTGGATCCAGACAAGCAGCC
